# Applying the improved stratigraphic modified Lorenz technique for dividing the highly heterogeneous clastic reservoirs into hydraulic flow units

**DOI:** 10.1038/s41598-023-47709-1

**Published:** 2023-11-22

**Authors:** Bassem S. Nabawy, Ahmed S. Mohamed, Awad A. Omran, Mostafa T. Mohamed

**Affiliations:** 1 Department of Geophysical Sciences, National Research Center, Cairo, Egypt; 2 Mining and Petroleum Department, Faculty of Engineering, Al-Azhar University, Qena, Egypt; 3 Geology Department, Faculty of Science, Assiut University, Assiut, Egypt; 4 Mining and Metallurgical Department, Faculty of Engineering, Assiut University, Assiut, Egypt

**Keywords:** Solid Earth sciences, Core processes, Geology

## Abstract

The present study applies the improved stratigraphic modified Lorenz (ISML) technique to divide the Matulla Formation in Muzhil Oil Field in the Gulf of Suez into some hydraulic flow units (HFUs) and to check the flow efficiency contribution of each hydraulic flow unit (HFU) to the total bulk flow capacity of the reservoir in 3 wells (Muzhil-4, 7, and 8). The output of the ISML plot is applied in integration with the vertical plot of the porosity (∅), permeability (k), and effective pore radius (R_35_) against depth to measure the efficiency of each HFU contribution to the total flow capacity of the Matulla reservoir, and to delineate the main attributor to the flow capacity. It is indicated that the Matulla sandstone reservoirs can be subdivided into 7 HFUs to the NW of the field, while it is subdivided into four and five HFUs in the center and to the SE of the field; i.e., its heterogeneity increases to the NW at Muzhil-7 well. On the other side, the best reservoir quality is assigned to the southeast at Muzhil-4 well (av. ∅ = 20.8%, av. k = 596.6 md, and R_35_ = 12.1 μm). The efficiency of the obtained HFUs was estimated and described both mathematically and graphically. Also, the measured porosity and permeability values indicate relatively low reservoir properties to the NW of the field. The reservoir heterogeneity is also measured using the Dykstra-Parsons technique which indicates extremely high heterogeneity (0.89 ≤ V ≤ 0.98).

## Introduction

The main target of the reservoir characterization process is to divide the reservoir sequence into promising and not promising hydraulic flow units (HFUs). It is primarily a complicated process in highly heterogeneous reservoirs. Heterogeneity is mostly attributed to the differential spatial distribution of the porosity and permeability due to either depositional or diagenetic factors. Discriminating the reservoir into some HFUs or units enabled a detailed reservoir characterization and in turn, enables an accurate prediction for the reservoir performance in the future. Discriminating the reservoir into HFUs as a function of depth is more favorable than its discrimination into reservoir rock types (RRTS) based on their lithology regardless their depths. On the other side, the reservoir discrimination into HFUs considers the depth; i.e., it is easily correlated with the well logging records.

Discriminating the reservoir sequence into flow units has been widely applied by many authors^1–12^. During the last decades, some techniques have been applied to divide the reservoir sequence into flow units. Among these, the stratigraphic modified Lorenz (SML) plot is widely applied^13–15^. Gunter et al.^13^ used this plot to divide and describe the reservoir sequence into non-conductive, conductive and super conductive zones^12^. It has been applied and verified by many authors over the last two decades^8,15–27^.

Nabawy^28^ improved the SML by adding more details on the classification ranks of the obtained HFUs and estimating the contribution efficiency of each HFU to the total flow capacity of the given reservoir. Two methods are applied in this ISML plot; graphically and mathematically to divide and describe the obtained HFUs as barriers, semi-barriers, baffles, semi-conductive, conductive, super conductive, fractured, and highly fractured.

Consequently, in the present study, the ISML technique is applied to the Lower Senonian Matulla Formation to divide it into HFUs considering the porosity–permeability-R_35_ vertical plot. Matulla Formation has been studied by many authors^23,24^. It is considered a 3rd order depositional sequence that is composed of some highstand, transgressive, and lowstand system tracts (HST, TST, and LST), representing accommodation space at the final basin filling stage, deeper marine setting, and shallow marine settings, respectively^20,21,25^. Matulla Formation has a highly prolific reservoir quality, particularly in its lower and middle units. The limestone and shale streaks of the Matulla Formation are known also as source rocks for kerogen types I/II and III with more than 2% TOC, high HI index (300–675), and low oxygen index (15–100)^26^. The Matulla Formation is considered also a highly heterogeneous reservoir in the central province of the Gulf of Suez. This will help greatly in realizing the heterogeneous nature of this reservoir and enables (1) its slicing into flow units, and (2) its detailed characterization.

## Theoretical background

The stratigraphic modified Lorenz (SML) plot is one of the most important techniques that are widely applied for the discrimination of a given reservoir sequence into some hydraulic flow unit (HFU). It is X–Y plot based on the porosity and permeability, which are multiplied by their representative bed thicknesses (h), and the obtained results are referred to the storage and flow capacities, respectively^2,13,25,27–31^. The cumulative storage and flow capacities for each point (in decimals) are calculated using the following mathematical models^29^.

1 $$ \emptyset \cdot h_{cumulative} = \frac{{\emptyset_{n} x h_{n} }}{{\sum \phi_{n} x h_{n} }} $$

2 $$k.{h}_{cumulative}=\frac{{k}_{n} x {h}_{n}}{\sum {k}_{n} x {h}_{n}}$$

where ∅ and k are the porosity and permeability, respectively; while h_cumulative_ is the cumulative corresponding thickness at the given point.

In the SML X–Y plot, the normalized ∅.h_cumulative_ (Eq. (1)) and k.h_cumulative_ for each plug sample are represented on the X and Y-axes. The storage and flow capacities data are arranged based on their corresponding depth in descending order in a way that the zero capacity point corresponds to the deepest point of the sequence while the unity capacity point corresponds to the highest top point in this sequence. Therefore, the depth of the stratigraphic sequence is taken into consideration, i.e., the sequence depth is respected^13,28,29^.

Then, the obtained storage capacity-flow capacity curve will be divided into some plateaus (line segments) of various slope ranges, separated from each other by inflection points. Each plateau represents a hydraulic flow unit (HFU) in the reservoir sequence; i.e., based on this plot the given reservoir can be divided into some HFUs (promising and not promising zones). The flow conductivity of these flow units can be deduced from the slope of its corresponding plateau. When the slope is close to the vertical 90°-line, the HFU is considered representative of a super conductive zone. On the other side, when the slope is near the horizontal 0°-line, the HFU is considered a barrier, whereas the in-between slope range represents conductive zones with various grades^3^, i.e., increasing the slope of a given plateau refers to increasing the ability of the HFU to transmit fluids^3,11,22,28^.

**Figure 1:**
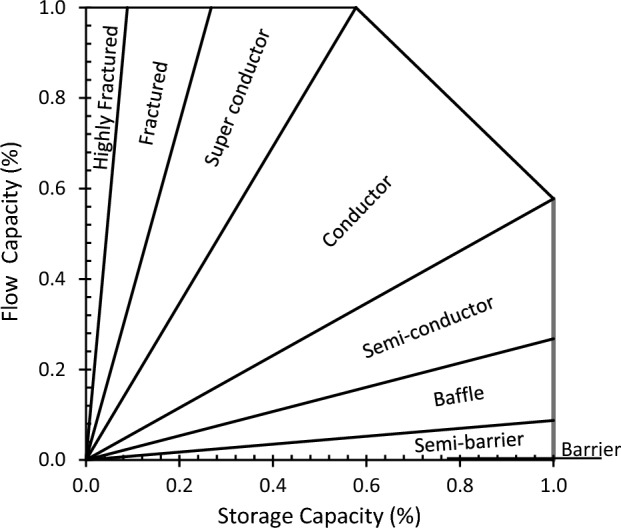
A simplified sketch presenting the ISML classification ranks the (HFUs) (Table 1)^28^. Nabawy (2021)^28^ proposed two methods to delineate the flow efficiency and contribution using the proposed ISML classification ranks; the graphical and the mathematical methods.

Nabawy^28^ introduced an improvement to this plot (ISML) by adding a more precise and fixed digital descriptive classification instead of a rough descriptive classification. He proposed eight classification ranks for the presented HFU based on their slope angles: (1) barrier (0°), (2) semi-barrier (0°–5°), (3) baffle (5°–15°), (4) semi-conductor (15°–30°), (5) conductor (30°–60°), (6) superconductor (60°–75°), (7) fractured (75°–85°), and (8) highly fractured (85°–90°) (Table 1, Fig. 1).

**Table 1:** The flow capacity classification ranks based on the ISML technique of Nabawy (2021)^28^.

Graphical method Slop angle (θ)	Description	Statistical method Tanθ	Efficiency rank
0°	Barrier	Tan θ < 0.017	Tight
1°–5°	Semi-barrier	0.017–0.087	
5°–15°	Baffle	0.087–0.268	
15°–30°	Semi-conductor	0.268–0.577	Poor
30°–60°	Conductor	0.577–1.732	Fair
60°–75°	Super conductive	1.732–3.732	Good
75°–85°	Fractured	3.732–11.43	Very good
85°–90°	Highly fractured	Tan θ > 11.43	Excellent

### Graphical method

In this method, the slope angle of each line segment, which represents a separate HFU, is measured. Then, based on this slope, the efficiency and the contribution of this HFU normalized to the total flow capacity of the given reservoir sequence can be estimated and ranked following the main eight ranks of Nabawy^28^ as mentioned in Table 1 and illustrated in Fig. 1. The steeper the slope of the line segment, the higher the efficiency of the corresponding HFU. Also, the longer the line segment, estimated on Y-axis, the more the contribution to the total flow contribution to the bulk reservoir flow ability. Therefore, this graphical (ISML) plot is considered a precise estimate of the ability of each HFU to transmit fluids and estimate the actual contribution of each HFU to the total flow capacity of the studied reservoir.

### Mathematical method

This method is based on estimating the slope of each HFU's line segment using a simple mathematical method. It processes each line segment as two ordered pairs representing the start (SC_1_, FC_1_) and the end (SC_2_, FC_2_) of this line segment. These ordered pairs represent the (x, y) coordinates of a storage capacity point (SC) on the X-axis and a flow capacity point (FC) on the Y-axis. The output of this method is numerical digits rather than angles as in the case of the graphical method (Table 1).

3 $$\mathrm{Tan}\theta =\frac{{\mathrm{FC}}_{2}-{\mathrm{FC}}_{1}}{{\mathrm{SC}}_{2}-{\mathrm{SC}}_{1}}$$

### Advantages of the ISML technique as a reservoir ranking method

Nabawy^28^ mentioned that dividing the reservoir sequence into HFUs using the ISML technique is preferred using the reservoir quality index (RQI) and the flow zone indicator (FZI) of Amaefule et al.^32^ and its classification ranks of Nabawy and El Sharawy^33^. He explained that by referring to the low porosity-high permeability reservoirs, which are considered good to excellent reservoirs (following the classification of the RQI and FZI of Nabawy and El Sharawy^33^ the reservoir quality is based on how much the porosity contributes to permeability rather than the magnitude of the permeability itself. Therefore, the ISML technique assigns the low porosity-high permeability reservoirs as fractured reservoirs, which is more logical than describing them by the RQI-FZI classification ranks.

El Sayed and El Sayed^9^ introduced a novel R_36_ which is very helpful to discriminate between dry (pore aperture size < 0.5 μm) and producing wells (pore aperture size > 0.5 μm or 5000 A°) in case of absent of porosity data. Model verification indicates a very close matching between R_35_ and Kr_36_ (the pore radius corresponding to 36% mercury saturation, calculated from permeability) vertical profiles in different oil fields of different geographic locations^9^.

## Available data and the applied methodology

The HFU efficiency in a given reservoir is a term that refers to the contribution of that HFU to the bulk flow capacity. It is considered a HFU quality or a HFUs’ efficiency measure.

**Figure 2:**
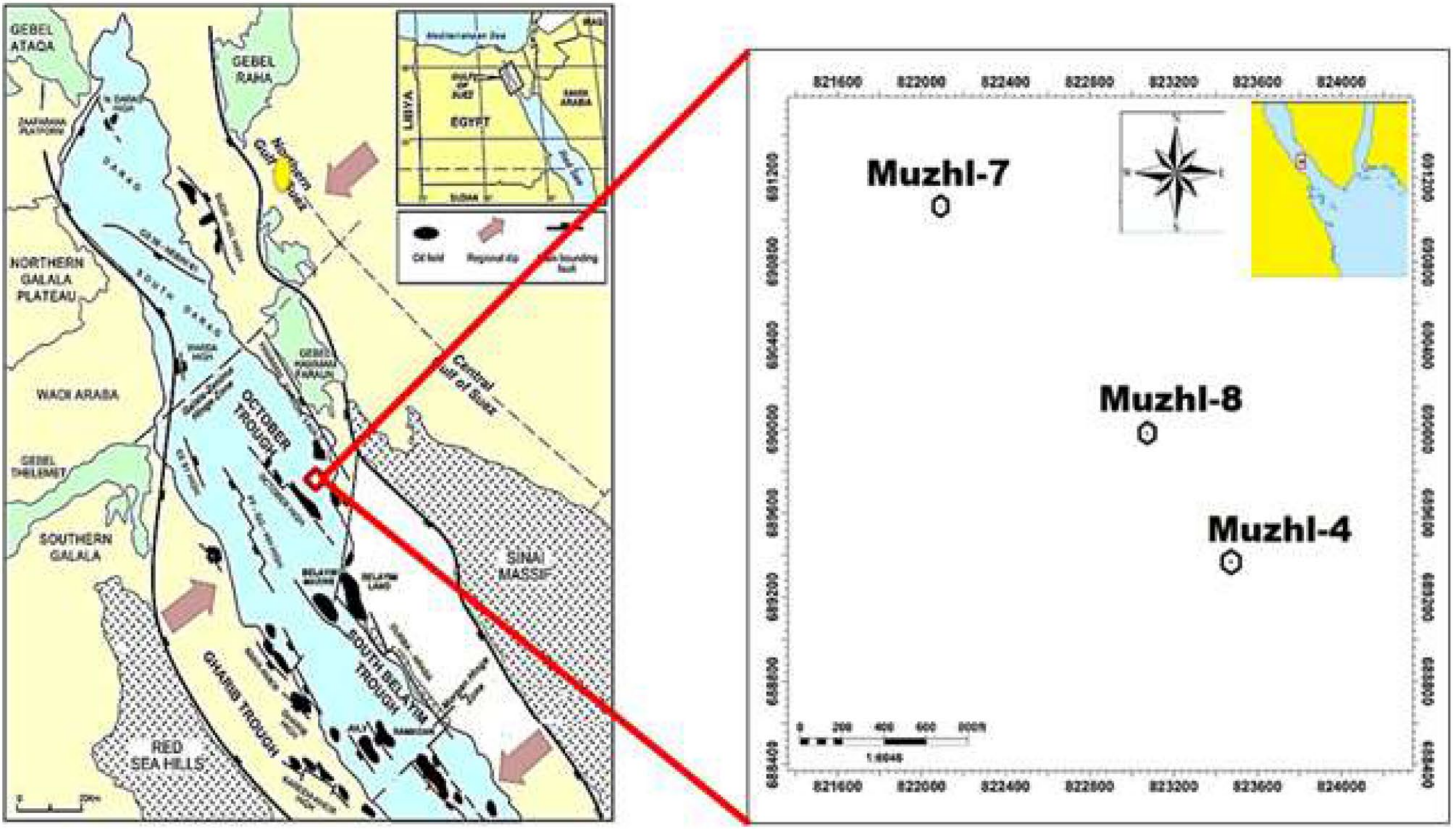
Location map of Muzhil oil field in the central Gulf of Suez^23^.

The Matulla Formation is encountered in Muzhil Field at depth intervals (11,280–11,636.9, 12,144–12,557.7, and 11,189.6–11,504.9 ft for Muzhil-4, 7 and 8 wells, respectively). These encountered intervals have been cored at depths 11,541–11,612, 12,355–12,542, and 11,395–11,457 ft, respectively). Porosity and permeability core data for a total number of 216 plug samples, selected representatively from the cored intervals of the clastic Matulla reservoirs in Muzhil-4, 7, and 8 wells in Muzhil Oil Field in the central Gulf of Suez Basin (Fig. 2), were released by PETROZENIMA Oil Company. The detailed measuring porosity and permeability methodology were published by many authors^8,11,22,28,29,34–36^.

The storage and flow capacities were estimated using Eqs. (1 and 2) as recommended by many authors^2,13,22,27–29,37–40^.

The ISML plot technique was applied to divide the reservoir sequence into HFUs in the three wells. The slope of the obtained line segments for each HFU was estimated graphically and its efficiency was then calculated and ranked following the efficiency classification ranks of Nabawy^28^. The slope of the HFUs in the ISML plot was also estimated mathematically using Eq. (3)^28^.

To explain and to check the main contributors to the estimated HFUs' efficiency, the effective pore throat diameter (R_35_) of Winland^41^ is calculated using the following equation.

4 $$ {\text{Log R}}_{{{35}}} = \, 0.{732 } + \, \left( {0.{588 } \times {\text{ log k}}} \right) \, {-} \, (0.{864 } \times {\text{ log}}\emptyset ) $$

The pore throat sizes are classified as follow^28^:

- Nano Pores: R_35_ < 0.1 μm
- Micro Pores: 0.1 μm ≤ R_35_ < 1.0 μm
- Meso Pores: 1.0 μm ≤ R_35_ < 10 μm
- Macro Pores: 10 μm ≤ R_35_ < 100 μm
- Mega Pores: 100 μm ≤ R_35_

Also, heterogeneity of the permeability values distribution among the various beds was estimated using the Dykstra-Parsons technique^38^; in which a cumulative frequency plot for k is obtained by arranging the permeabilities of the studied plugs in descending order. Permeability values were then estimated at 50% and 84.1% probabilities and the heterogeneity variation (V) was estimated as follows:

5 $$\mathrm{V}=\frac{{\mathrm{k}}_{50}-{\mathrm{k}}_{84.1}}{{\mathrm{k}}_{50}}$$

To introduce a ranking measure for the reservoir heterogeneity, the heterogeneity classification ranks of El Sharawy and Nabawy^15^ were applied describing the reservoir heterogeneity as follows^8,38–40^:

- Extremely heterogeneous, V = 0.75–1.00;
- Highly heterogeneous, V = 0.50–0.75;
- Moderately heterogeneous reservoir, V = 0.25–0.50;
- Slightly heterogeneous, V = 0.10–0.25; and
- Homogeneous, V < 0.10.

The efficiency contribution percentage of each HFU is estimated as its contribution percentage to the total flow capacity as estimated from Eq. 2 (the summation of all permeability values measured for a given thickness).

6 $$\mathrm{HFU\, effeciency\, contribution\,}(\mathrm{\%})=\frac{{\mathrm{FC}}_{2}-{\mathrm{FC}}_{1}}{100}$$

### Ethics approval

We hereby confirm that the present research has no materials that need to be approved ethically. It is just processed geological data.

## Results

Based on the core description data, the plug samples of the Matulla reservoir in Muzhil Field are primarily composed of alternated streaks of grey fine to medium-grained, well-sorted, and well-cemented sandstone. Cement is primarily calcareous; sometimes with silty patches and glauconite pellets. The Matulla sandstone is sometimes cemented by argillaceous or dolomitic cement. Also, some weakly cemented and fine to very fine-sandstone steaks are present. Heterogeneity of the mineral composition of the Matulla reservoir is highly considered due to variation in the grain size from very fine to medium-grained, cementation by various cements (calcareous, argillaceous, and/or dolomitic), grains are sometimes tightly or weakly cemented; additional mica and glauconite content is also present. The porosity of the studied samples is highly variable from 1.5% in Muzhil-7 well to 29.0% in Muzhil-4 well. Muzhil-7 well is characterized by the lowest average porosity (10.9%, Table 2). Also, permeability varies intensively from 0.007 md and 0.012 md in Muzhil-7 and Muzhil-8 wells to 3001 md in Muzhil-4 well. The highest average permeability was estimated for the reservoir sequence in Muzhil-4 well (av. k = 596.6 md), while the lowest av. k was assigned for the Muzhil-7 well (1.01 md, Table 2). Also, the highest average R_35_ is assigned to the Muzhil-4 well (av. R_35_ = 12.1 μm, macro pores, Table 2), while the lowest R_35_ is recorded for the Muzhil-7 well (av. R_35_ = 0.49 μm, micro pores, Table 2).

**Table 2:** The petrophysical and reservoir quality parameters and the pore sizes of the Matulla Formation in the various wells of Muzhil Field, central Gulf of Suez, based on the conventional core data analysis.

Well	N		$\emptyset$_He_ (%)	k (md)	R_35_ (μm)	Pore sizes	V
Muzhil-4	62	Min	12.1	0.170	0.2	Micro	0.96
		Max	29.0	3001	35.7	Macro	
		Mean	20.8	596.6	12.1	Macro	
Muzhil-7	101	Min	1.5	0.007	0.04	Nano	0.89
		Max	21.4	22.00	3.66	Meso	
		Mean	10.9	1.01	0.49	Micro	
Muzhil-8	53	Min	3.30	0.012	0.05	Nano	0.98
		Max	28.1	358.0	11.25	Macro	
		Mean	17.0	82.2	3.50	Meso	

∅_He_ is the helium porosity, k is the air permeability, R_35_ is the effective pore radius of Winland^41^, and V is the permeability heterogeneity of Dykstra-Parsons^38^.

Using the Dykstra-Parsons technique^37,38^ indicates that the Matulla formation is highly heterogeneous with the heterogeneity index V varying from 0.89 up to 0.98 in the various wells. The Matulla Formation is divided into 4 HFUs in Muzhil-8 well, 5 HFUs in Muzhil-4 well, and 7 HFUs in Muzhil-7 well.

## Discussion

### Delineating the reservoir heterogeneity

#### Permeability attributes

**Figure 3:**
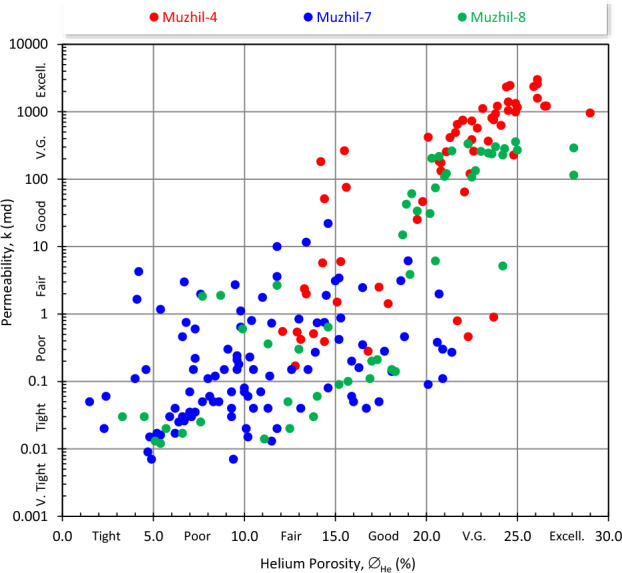
Plotting the permeability as a function of the Helium porosity (∅_He_) of Muzhil 4, 7, and 8 wells.

The reservoir heterogeneity refers to variation of the reservoir properties from depth to depth; i.e., from bed to bed. The heterogeneity grade or homogeneity can be realized by plotting the permeability (k) values of the studied plugs versus their corresponding porosity (∅) values as shown in Fig. 3, where k and ∅ of the Muzhil wells were presented. This plot indicates a high scattering of the presented data, especially for samples of the Muzhil-8 well. It is revealed that samples of Muzhil-4 well are characterized by the best reservoir quality (mostly very good to excellent ∅ and k); samples of Muzhil-7 well are characterized by the least reservoir parameters (tight to good ∅, and tight to fair k), while variation in samples of the Muzhil-8 well is high representing high, medium and low reservoir parameters (tight to very good k and ∅) (Fig. 3). This scattering means that no reliable best-fit lines between porosity and permeability can be obtained for the various wells.

#### Permeability variation

The permeability heterogeneity (V) is the main indicator of the reservoir heterogeneity it is an indication of the variation of permeability from sample to sample, i.e., from bed to bed. Therefore, the homogeneous clastic reservoirs are characterized by clustered permeability data indicating a homogeneous to slightly heterogeneous nature, while the intercalated reservoirs, like the case of Matulla reservoirs, are characterized by scattered permeability data delineating a highly heterogeneous nature of these reservoirs.

**Figure 4:**
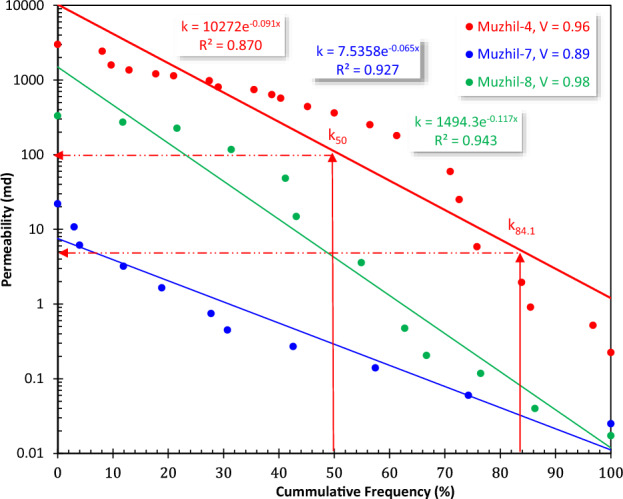
Applying the Dykstra–Parsons technique^38^ to check the permeability heterogeneity of the Matulla reservoir in Muzhil Field.

For the present study, the permeability heterogeneity is checked by applying the Dykstra-Parsons technique^38^. This plot indicates an extremely heterogeneous reservoir nature, where V varies from 0.89 for Muzhil-7 well to 0.98 for Muzhil-8 well (Fig. 4). This achievement is in accordance with that achieved from the porosity–permeability (Fig. 3) that the permeability data of Muzhil-8 is highly scattered and characterized by a wide range of variation (0.012 ≤ k ≤ 358, Table 1).

Thereby, due to the extremely heterogeneity nature of the Matulla Formation, dividing its reservoir sequence in the Muzhil wells into HFUs should be applied using the SML plot of Gunter et al.^13^ and its modification by Nabawy^28^ to describe and estimate accurately the efficiency and the contribution of each HFU individually to the bulk reservoir flow capacity as follows.

### Reservoir discrimination using the ISML plot

**Figure 5:**
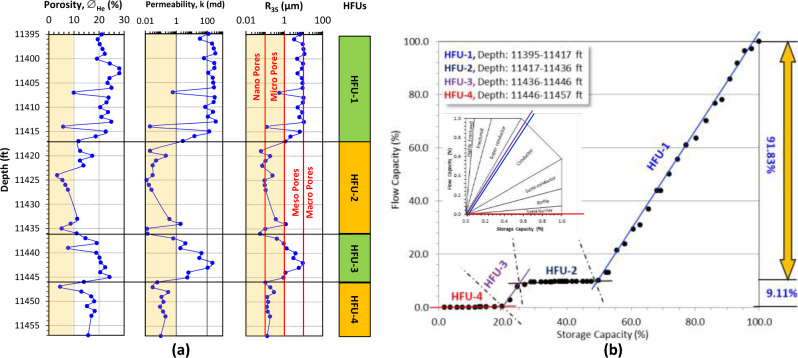
Discriminating the clastic Matulla Formation in Muzhil-8 well into HFUs using: (**a**) Conventional vertical plotting for core data (green-shaded HFUs are promising, while the dark orange-shaded HFU is not promising), the light orange shadow on the porosity, permeability and R_35_ tracks refer to the applied cutoff values (10% for ∅, 1.0 md for k, and 1.0 μm for R_35_), and (**b**) improved stratigraphic Lorenz model (ISML).

**Figure 6:**
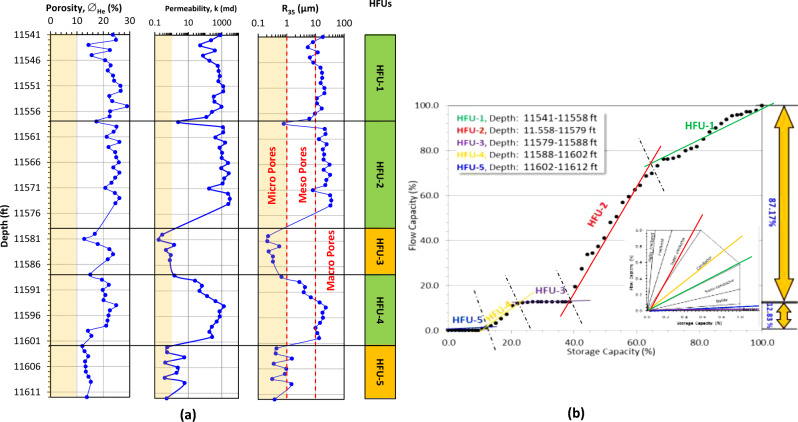
Discriminating the clastic Matulla Formation in Muzhil-4 well into HFUs using: (**a**) conventional vertical plotting for core data (green-shaded HFUs are promising, while the dark orange-shaded are not promising), the light orange shadow on the porosity, permeability and R_35_ tracks refer to the applied cutoff values (10% for ∅, 1.0 md for k, and 1.0 μm for R_35_), and (**b**) improved stratigraphic Lorenz model (ISML).

**Figure 7:**
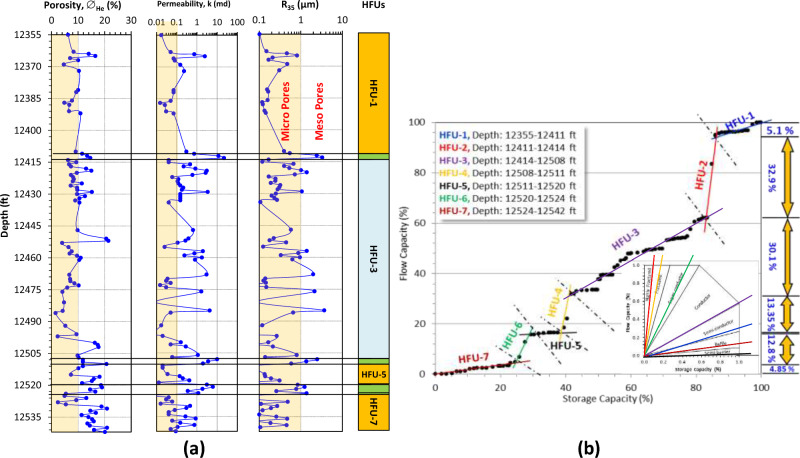
Discriminating the clastic Matulla Formation in Muzhil-7 well into HFUs using: (**a**) conventional vertical plotting for core data (green-shaded HFUs are promising, the dark orange-shaded are not promising, while the blue-shaded HFU represents alternation between promising and not promising zones), the light orange shadow on the porosity, permeability and R_35_ tracks refer to the applied cutoff values (10% for ∅, 1.0 md for k, and 1.0 μm for R_35_), and (**b**) improved stratigraphic Lorenz model (ISML).

Applying the ISML plot for the Matulla reservoir sequence in the Muzhil wells is required due to the extremely high heterogeneity as indicated in Fig. 3b. It helped in discriminating the sequence into seven HFUs. Accompanying this plot with the vertical plot of the reservoir parameters including the porosity, permeability, and the R_35_ supports this achievement and delineates the main reasons for this discrimination (Figs. 5, 6, 7).

Also, this plot estimates the contribution of each HFU in the studied wells and follows the lateral change of the Matulla Formation from well to well. It is indicated that the Matulla Formation is divided into 7 HFUs in Muzhil-7 to the NW only, while the number of HFUs decreases to the center at Muzhil-4 (4 HFUs), and toward the SW at Muzhil-8 (5 HFUs). In Muzhil-8 well at the center of the Muzhil Field (Fig. 2), the Matulla reservoir consists of an alternation of two promising HFUs and other two not promising HFUs (Fig. 5a). It is indicated that porosity, permeability, and R_35_ of the HFU-2 (19 feet), and HFU-4 (11 feet) are less than the cutoff values, i.e., they are not promising. Applying the ISML plot indicates that these two HFUs (represented by the black and red plateaus in Fig. 5b) are considered semi-barrier (Fig. 5b) with a total contribution of less than 1.0% for the bulk reservoir flow capacity (Table 3). The HFU-1 (22 feet) and HFU-3 (10 feet) are the most promising in Muzhil-8 well (represented by the blue and violet segments in Fig. 5b). Following the efficiency classification of Nabawy^28^, they are considered conductive HFUs with a total efficiency contribution 99.1% of the bulk flow capacity (Table 3). These two HFUs are characterized by permeability of more than 100 md for the HFU-1 and mostly more than 10 md for the HFU-3. This can be attributed to meso pore sizes of these HFUs with R_35_ reaching primarily to 10 μm for HFU-1 and touching the 10 μm line with one peak (Fig. 5a).

**Table 3:** Discriminating the Matulla reservoir in Muzhil Oil Field into some hydraulic flow units (HFUs).

HFUs	Muzhil-4 well	Muzhil-7 well	Muzhil-8 well
HFU-1	Super-conductor (23.78%)	Semi-conductor (5.1%)	Conductor (89.99%)
HFU-2	Conductor (63.49%)	Super conductive (32.9%)	Semi-barrier (0.61%)
HFU-3	Semi-barrier (0.74%)	Conductor (30.1%)	Conductor (9.11%)
HFU-4	Conductor (11.94%)	Super conductive (13.35%)	Semi-barrier (0.29%)
HFU-5	Semi-barrier (0.05%)	Semi-barrier (0.9%)	
HFU-6		Super-conductor (12.8%)	
HFU-7		Baffle (4.85%)	

The HFU's quality rank and its contribution percentage to the total flow capacity of the reservoir sequence are also indicated (quality ranks are based on the classification ranks in Table 1, Nabawy^28^).

To the southeast, Matulla Formation in Muzhil-4 well is characterized by the best reservoir properties. It is divided into 5 HFUs, 3 promising (HFU-1 (17 feet), HFU-2 (21 feet thickness), and HFU-4 (14 feet thickness thickness)), and two not promising (HFU-3 (9 feet thickness), HFU-5 (10 feet thickness)) (Fig. 6). It seems that the HFU-1 in Muzhil-8 well is subdivided to the southeast into two HFUs due to the development of barrier streak of 1 feet thickness (Fig. 6a). However, the reservoir quality of these two units highly increased in Muzhil-4 with porosity and permeability more than 20% and 1000 md, respectively (Fig. 6a). These two units are considered conductive and super conductive (Fig. 6b); respectively, with a total contribution of 87.27%.The efficiency of these two HFUs is attributed to their macro pore sizes (R_35_ > 10 μm, Fig. 6a). Downward, the HFU-3 and HFU-5 are characterized by micro pore spaces (R_35_ < 0.1 μm), so their permeability values are less than the cutoff value (1.0 md, Fig. 6a). Consequently, these two HFUs are acting like semi-barriers (blue and black line segments, Fig. 6b) with a total contribution to the bulk flow capacity equals 0.79% (Table 3).

Toward the NW in Muzhil-7 well, the Matulla reservoir can be divided into seven HFUs; three promising HFUs (HFU-2 (3 feet thickness), HFU-4 (3 feet thickness), and HFU-6 (4 feet thickness)), intercalated with 3 not promising zones (HFU-1 (56 feet), HFU-5 (9 feet thickness), and HFU-7 (18 feet thickness), and one spiky HFU-3 (94 feet, Fig. 7a). The total thickness of the promising zones in this well doesn't exceed 10 feet in total, i.e., the reservoir quality decreases in this well to the NW of the field. However, some additional interbeds and laminas are considered promising in HFU-3 (Fig. 7a). The promising HFU-4 and HFU-6 are characterized by permeability between 1 and 10 md due to their meso pore sizes, while upward the permeability of the HFU-2 exceeds 100 md in some parts. On the other side, the porosity and permeability of the not promising units are less than the cutoff values, except for one or two peaks in HFU-1 and HFU-5. This could be attributed to their micro pore sizes (R_35_ < 1.0 μm). The HFU-3 is a spiky unit with many not promising zones of poor porosity (∅ < 10%) but has fair to good permeability (1–10 md, Fig. 7a).

Concerning the ISML plot (Fig. 7b), it is indicated that HFU-5 is considered semi-barrier (black segment, Fig. 7b) with 0.9% efficiency contribution to the total flow capacity (Table 3). The promising HFUs are considered conductive to super conductive units (brown, orange, and green segments, Fig. 7b) with 59.05% as a total contribution to the total flow capacities (Table 3). The spiky HFU-3 unit, which is characterized by micro to meso pores, contributes by 30.1% to the total flow capacity; it is considered a conductive unit (Table 3, Fig. 7b). The not promising HFU-1 is considered semi-conductor of 5.1% contribution, the HFU-5 is considered semi-barrier of 0.9% contribution, while the HFU-7 is considered baffle 4.85% (Table 3, Fig. 7b).

In general, the integration between the ISML technique and the vertical plot for the reservoir parameters helps in dividing the reservoir into some HFU and measuring the efficiency of each HFU to contribute to the total flow capacity of the reservoir.

Though the Dykstra-Parsons technique^38^ indicates that the permeability heterogeneity (V = 0.89) is the least in Muzhil-7 well in comparison to the other wells, the integration between the ISML technique and the porosity–permeability-R_35_ plot indicates that it is characterized by more heterogeneity than the other wells. This contradiction can be explained by the fact that the Dykstra-Parsons technique^38^ concerned with the variation range of the permeability values, i.e., the wider the variation range the higher the permeability heterogeneity. On the other side, the ISML plot is based on graphical discrimination of the reservoir into HFUs based on their flow and storage capacities, which means that the presence of only one promising HFU with a wide range of k will be characterized by extremely high heterogeneity based on the Dykstra-Parsons technique^38^, which is not realistic. On the other side, the presence of many alternated promising and not promising HFUs with a limited range of k variation, like the case of the Matulla reservoir in the Muzhil-7 well, is considered less heterogeneous based on the Dykstra-Parsons technique^38^. Therefore, applying the ISML plot is considered more reliable in delineating the heterogeneity of the studied reservoirs than the Dykstra-Parsons technique^38^.

## Conclusions

- Plotting porosity versus the permeability of the Matulla reservoir in Muzhil Oil Field indicates that the porosity–permeability data are scattered. This scattering was checked for the various wells in the field using the Dykstra-Parsons technique indicating extremely high heterogeneity nature for the Matulla reservoir (V ≥ 0.89).
- This high permeability scattering can be attributed to the presence of various pore radii with a wide range of the effective pore throat radius (R_35_) which is the main attributor to the permeability of the studied samples.
- Due to the high heterogeneity of the Matulla reservoir, it is subdivided into seven hydraulic flow units (HFUs) to the NW of the field while subdivided into five HFUs to the SE of the field.
- The assigned HFUs are discriminated into not promising HFUs (semi-barrier and baffle HFUs) alternated with promising HFUs (conductor and super conductor HFUs).
- Integrating the ISML plot with the porosity–permeability-R_35_ vertical plot delineates the relatively high heterogeneity of the Matulla Formation. Also, the net result of the flow capacity estimations showed the contribution of each HFU to the total flow capacity.

## Limitations

- The applied ranks are based on oil-bearing reservoir real data, so their extension to gas-bearing reservoirs should be applied carefully.
- The mentioned ranks are relative ranks to compare the efficiency contribution of each HFU within the same reservoir and should not be applied in a comparative study between different reservoirs.

## Data Availability

Due to the confidentiality agreement of the present data, we do not have permission to share our data. It is just permission to process and present our concepts and interpretation of the released data. For any declarations, the corresponding author, Bassem Nabawy, will be responsible for response.
